# An Improved Beta Burst Extraction for Chip-Based Deep Brain Stimulation With Real-Time Model Updating

**DOI:** 10.1109/OJEMB.2026.3695567

**Published:** 2026-05-25

**Authors:** Yi-Huan Ou-Yang, Hsiao-Chun Lin, Chi-Wei Huang, Chung-Yu Wu, Ming-Dou Ker, Chen-Yi Lee

**Affiliations:** Institute of ElectronicsNational Yang Ming Chiao Tung University 34914 Hsinchu City 30010 Taiwan; Biomedical Electronics Translational Research CenterNational Yang Ming Chiao Tung University 34914 Hsinchu City 30010 Taiwan; Biomedical Electronics Translational Research CenterNational Yang Ming Chiao Tung University 34914 Hsinchu City 30010 Taiwan; the Biomedical Electronics Translational Research CenterNational Yang Ming Chiao Tung University 34914 Hsinchu City 30010 Taiwan; Amazing Neuron Corporation Hsinchu 30273 Taiwan; Institute of ElectronicsNational Yang Ming Chiao Tung University 34914 Hsinchu City 30010 Taiwan; the Biomedical Electronics Translational Research CenterNational Yang Ming Chiao Tung University 34914 Hsinchu City 30010 Taiwan; Amazing Neuron Corporation Hsinchu 30273 Taiwan; Institute of ElectronicsNational Yang Ming Chiao Tung University 34914 Hsinchu City 30010 Taiwan

**Keywords:** Adaptive neuromodulation, beta burst extraction, deep brain stimulation, implantable medical devices, real-time signal processing

## Abstract

*Goal:* Existing beta burst detection algorithms for closed-loop deep brain stimulation (DBS) are computationally complex, limiting their use in implantable devices. We aimed to develop an improved beta burst extraction algorithm for chip-based DBS devices with real-time model updating. *Methods:* Building on an established beta burst detection method, we proposed a sliced mechanism for peak frequency finding and modified burst extraction for information sharing with real-time model updating. *Results:* Testing on rat electrocorticographic (ECoG) recordings showed that the proposed algorithm maintains a strong correlation ( 0.89 0.06) with the conventional method, with a 53.3 reduction in computational complexity for peak frequency finding. *Conclusions:* Integrating this improved beta burst detection into chip-based DBS devices represents a key algorithmic advancement toward adaptive neuromodulation therapies. The strong correlation and reduced complexity validate our proposal for real-time neural biomarker tracking, facilitating hardware and chip implementation, and advancing the development of implantable systems.

## I. Introduction

Parkinsons disease (PD) is a progressive neurodegenerative disorder characterized by abnormal basal ganglia oscillations, particularly in the beta band (1335 Hz), which are associated with motor impairments including bradykinesia and rigidity [1], [2]. Deep brain stimulation (DBS) of the subthalamic nucleus (STN) has emerged as an effective treatment for advanced PD motor complications [3], [4]. However, conventional DBS systems deliver continuous stimulation with fixed parameters, resulting in suboptimal efficacy and unnecessary energy expenditure [5], [6]. Adaptive or closed-loop DBS strategies have been proposed to address these limitations by dynamically adjusting stimulation based on real-time neural biomarkers, including amplitude-adaptive and phase-locked control schemes [5], [6], [7], [8], [9].

Transient increases in beta-band power, termed beta bursts, have gained substantial interest due to their stronger correlation with motor symptoms than sustained beta activity [10]. Real-time detection of beta bursts provides a promising mechanism for event-triggered neuromodulation. Several burst detection algorithms have been developed for research, including amplitude envelope thresholding and burst-rate quantification [11], [12], [13], [14]. However, most approaches rely on long-window spectral analysis or offline statistical thresholding [10], which are computationally intensive and require substantial memory resources, making them unsuitable for long-term deployment in implantable devices.

Realizing closed-loop DBS for chronic implantation imposes stringent power consumption, memory usage, and real-time adaptability requirements. Compared to desktop processors, implantable systems are fundamentally constrained by limited battery capacity and computational resources [15], [16], [17]. While recent advancements in fully integrated closed-loop neuromodulation systems-on-chip (SoCs), such as NeuralTree, have demonstrated the feasibility of addressing these constraints through on-chip signal processing and classification [18], [19], the design of computationally efficient and adaptive beta burst detection algorithms remains a critical challenge. Consequently, beta burst detection methods must be carefully optimized to minimize their hardware footprint while maintaining robustness and accuracy under implantable device constraints. Additionally, dynamic adaptation to signal nonstationarities, such as shifts in beta frequency due to medication or behavioral changes, remains a major challenge in existing implementations that rely on static filter parameters and fixed thresholds [20]. These challenges are exemplified in clinically deployed research platforms such as the Medtronic Summit RC+S, where adaptive stimulation relies on predefined biomarker characteristics and configurable on-board spectral processing parameters. In such systems, exhaustive parameter tuning in patients is often impractical [21], underscoring the importance of algorithmic approaches that enable autonomous, real-time parameter adaptation under stringent device constraints.

The primary motivation for this work stems from the critical gap between the clinical promise of closed-loop DBS and the practical limitations of current implementation approaches. While beta burst detection has been validated as an effective biomarker for adaptive neuromodulation, existing algorithms remain too computationally complex for integration into clinically viable implantable systems. Conventional methods power consumption, memory requirements, and processing latency fundamentally limit their applicability in battery-operated neural implants.

This study aims to bridge this translational gap by developing a computationally improved beta burst detection algorithm for chip-based, real-time implementation in implantable DBS systems. Our primary objective is to substantially reduce computational complexity and memory requirements while maintaining sufficient accuracy for effective closed-loop DBS. Additionally, we seek to enable real-time model updating so the system can adapt to neural changes without offline recalibration.

The specific goals of this research are threefold: first, to develop an improved peak frequency estimation method that reduces computational burden while preserving detection sensitivity; second, to implement a frequency-domain burst detection approach that enables efficient resource sharing; and third, to establish an online parameter adaptation framework that eliminates the need for large historical data buffers while maintaining robust threshold estimation. Through these innovations, we aim to provide a computationally efficient framework for implementing adaptive spectral-domain feature extraction within the stringent resource constraints of implantable neuromodulation systems.

## II. Materials and Methods

### A. Overview of the Established Beta Burst Detection Method Used As a Foundation

Our work builds upon the foundational beta burst detection methodology for closed-loop DBS in Parkinsons disease [10]. Beta bursts within the 1335 Hz frequency range are critical biomarkers that correlate strongly with motor symptoms [10], [11]. The conventional approach comprises several key steps:

#### 1) Signal Filtering

Local field potential (LFP) recordings are band-pass filtered at the individualized beta peak 3 Hz (e.g., 1723 Hz for a 20 Hz peak) to isolate relevant oscillations and minimize adjacent band contributions [22].

#### 2) Amplitude Envelope Generation

The filtered signal is rectified and smoothed with a 400-ms moving average window to generate an amplitude envelope, reflecting time-varying beta power.

#### 3) Amplitude Thresholding

Bursts are periods where the amplitude envelope exceeds an adaptive threshold, typically the 75th percentile of the amplitude envelope distribution [10].

#### 4) Burst Characterization

Bursts are characterized as consecutive periods above the threshold, enabling analysis of features like rate, duration, and amplitude, correlated with clinical variables.

Though effective in practice, the reliance on continuous long-window processing imposes high computational and memory demands, making the method unsuitable for power-constrained implantable DBS systems and highlighting the need for a streamlined alternative.

### B. Proposed Sliced Computation Algorithm for Peak Frequency Finding

We propose a sliced computation algorithm with mode-based frequency selection for peak frequency estimation to address existing methods computational and memory constraints in implantable devices. Unlike conventional long-window Fast Fourier Transform (FFT) approaches, our algorithm segments long-duration LFP recordings into shorter, non-overlapping one-second windows. FFT is performed independently on each segment. The peak frequency is then determined as the mode of all analyzed segment-wise frequency maxima, constrained to the physiologically relevant beta band (1335 Hz).

This sliced computation approach significantly reduces the computational burden. Let N be the total data length and L the segment length, such that *M* *N/L* represents the number of segments. While conventional methods exhibit a complexity of *O*(*N* log_2_ *N*), our algorithm performs *M* independent FFTs, each with *O*(*L* log_2_ *L*) complexity. The total computational complexity is therefore *O*(*N* log_2_ *L*). Given *L* *N*, this yields substantial computational reduction.

Furthermore, memory requirements are greatly minimized. The proposed sliced computation operates on fixed-length segments of size *L*, requiring storage of only a single data segment and its associated FFT working buffer at any given time. In contrast, conventional peak frequency estimation based on long-window spectral analysis must buffer the entire analysis window of length *N*, resulting in substantially higher memory usage. Accordingly, while the computational complexity reduction is governed by the logarithmic term (log_2_ *N* to log_2_ *L*) as described above, the memory footprint is reduced directly with the FFT length, from *O*(*N*) to *O*(*L*). This segment-wise processing strategy enables efficient peak frequency tracking with a compact memory footprint, making it well suited for resource-constrained systems. Additionally, the one-second segment length provides a frequency resolution of 1 Hz, which is adequate for tracking clinically relevant variations in beta-band peak frequency. By localizing spectral estimation to individual segments, transient artifacts or short-duration disturbances are confined to a single window, thereby improving the stability of peak frequency selection without requiring long analysis windows.

### C. Modified Extraction Operation for Information Sharing With Model Updating

To support real-time model updating and reduce computational redundancy, beta burst extraction is performed in the frequency domain, allowing direct reuse of FFT results from the peak frequency estimation step. For each FFT window, beta burst power (P_beta_) is calculated by integrating the power spectral density (PSD) over a narrow band centered on the mode-selected peak frequency (fpeak):

\begin{equation*}{{P}_{beta}} = \sum\limits_{f = {{f}_{peak}} - \Delta f}^{{{f}_{peak}} + \Delta f} {PSD\left( f \right)} \tag{1} \end{equation*}

where *f* 3 Hz.

**Fig. 1.**
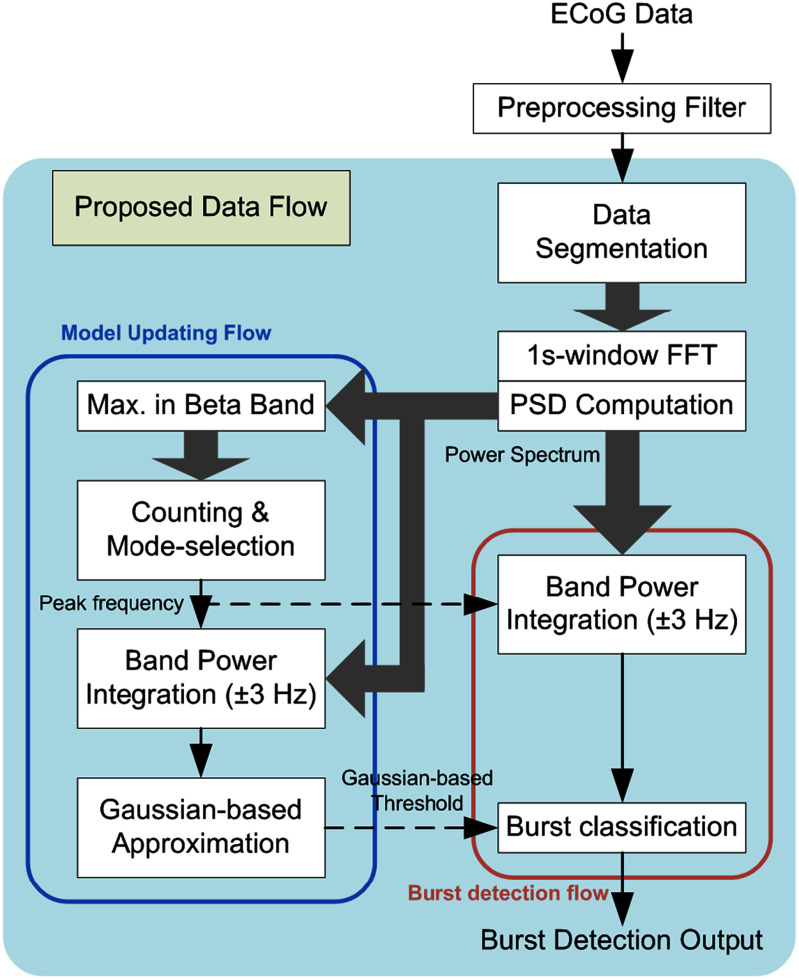
Algorithmic data flow of the proposed frequency-domain beta burst detection and model updating framework. Fig. 1. Algorithmic data flow of the proposed frequency-domain beta burst detection framework. Electrocorticographic (ECoG) signals are first processed by a front-end preprocessing filter and segmented into non-overlapping 1-s windows for FFT-based power spectral density (PSD) computation. The resulting spectral representation is shared between two processing paths. The burst detection flow (right, red region) integrates band power within a 3 Hz range around the estimated peak frequency and classifies burst events using an adaptive threshold. In parallel, the model updating flow (left, blue region) tracks the dominant peak frequency within the beta band (1335 Hz) using a mode-based counting scheme and updates statistical parameters via a streaming Gaussian-based approximation. Dashed arrows indicate parameter updates rather than sample-wise signal paths. This figure illustrates the algorithmic processing flow and does not imply a specific hardware or silicon-level implementation.

The proposed framework adopts a dual-flow processing scheme, in which burst detection and model updating are executed in parallel at the algorithmic level. Specifically, burst events are detected in real time using the current adaptive parameters, while model parameters such as the dominant peak frequency and statistical thresholds are updated continuously based on streaming spectral information. The FFT-based power spectral density computation is shared between the two processing flows, avoiding duplicated spectral analysis and large historical data buffers. An overview of the algorithmic data flow is illustrated in Fig. 1.

We adopt an online Gaussian-based approximation to eliminate the need for historical buffers to replace conventional percentile-based threshold estimation. This Gaussian-based formulation leverages the fact that frequency-domain beta power estimates are obtained by aggregating spectral energy over multiple time samples within each analysis window. Under this aggregation, the resulting power distribution can be reasonably approximated as Gaussian, consistent with the Central Limit Theorem [23, p. 164]. Consequently, a parametric threshold model can be employed to enable robust online estimation without requiring long-duration signal history. The 75th percentile threshold is approximated as:

\begin{equation*} Threshold = \mu + 0.675{\mathrm{ }}\sigma \tag{2} \end{equation*}

where is the running mean of the frequency-domain power values, and 0.675 corresponds to the z-score at the 75th percentile under a standard normal distribution [24]. Standard deviation () is computed online via the bias-corrected sample variance formula (Bessels correction) [23, p. 27], [25]:

\begin{equation*} Var\left( x \right) = \frac{n}{{n - 1}}\left( {E\left[ {{{X}^2}} \right] - {{{\left( {E\left[ X \right]} \right)}}^2}} \right) \tag{3} \end{equation*}

Both the mean (E[x]) and the second moment (E[x^2^]) are updated incrementally using streaming data. This streaming-based adaptation maintains robust real-time threshold estimation, eliminating offline recalibration and reducing memory overhead, which is critical for implantable systems.

### D. Evaluation Methodology

#### 1) Animal Model and Recording Protocol

Adult male Sprague-Dawley rats (n 9; 220260 g) received unilateral 6-OHDA lesions (3 g/L, 3 L) into the right medial forebrain bundle (AP 4.2 mm, ML +1.8 mm, DV 7.6 mm from bregma; IACUC approval: NCTU-IACUC-110033). Lesion efficacy was verified via apomorphine testing (>6 ipsilateral rotations/min over 20 minutes) three weeks post-injection. Cortical screw electrodes were implanted over the right motor cortex with a cerebellar reference. Electrocorticographic (ECoG) recordings from the motor cortex were employed in this study to evaluate the proposed algorithm. Cortical beta oscillations exhibit strong coherence with STN activity under Parkinsonian conditions and have been widely reported as correlates of pathological beta synchronization in both human and animal models [7], [26]. Compared to depth electrode recordings, cortical ECoG provides stable, high-fidelity signals that are less susceptible to electrode displacement or long-term recording artifacts. Importantly, the proposed algorithm is grounded in general signal-processing principles rather than a specific recording modality, allowing the computational framework to be readily extended to STN-LFP or other neural biomarkers in integrated DBS systems. After one week of recovery, ECoG signals were recorded from awake, freely moving animals using differential amplification (gain: 1000, 0.1500 Hz bandpass) and digitized at 1 kHz. Visually selected 90-second low-artifact periods were extracted from a single 3-minute recording session per animal and bandpass filtered (550 Hz) to suppress noise. Visual documentation of the electrode arrangement and representative neural signal traces are provided in the Supplementary Materials.

#### 2) Evaluation Metrics

Performance was evaluated across four dimensions to validate real-time operation and detection accuracy:

##### a) Peak frequency accuracy

Absolute differences in beta peak frequencies between the sliced computation and long-window FFT methods were quantified.

##### b) Computational efficiency

FFT-related computational complexity (*O*(*N* log_2_ *L*) vs. *O*(*N* log_2_ *N*)) and memory usage were assessed using 256-second datasets at 1 kHz and downsampled 128 Hz configurations optimized for hardware deployment.

##### c) Burst amplitude consistency

Spearman correlation assessed agreement between frequency-domain and time-domain amplitude measures, using standardized 1-second non-overlapping windows.

##### d) Threshold estimation and classification

Absolute deviation from target percentiles validated the Gaussian-based threshold approximation. Detection agreement was quantified using Cohens kappa, and F1-scores evaluated event-level classification performance.

All analyses were conducted in MATLAB (MathWorks Inc., Natick, MA) for reproducibility and benchmark comparison.

## III. Results

### A. Accuracy of Peak Frequency Estimation

The proposed mode-based sliced computation algorithm demonstrated strong concordance with the conventional long-window FFT method across all LFP datasets (n 9), with both approaches consistently identifying the dominant beta frequency within the 1335 Hz range. Absolute differences were within 1 Hz for 88.9 of recordings (8 out of 9), with a mean absolute difference of 0.61 0.64 Hz (range: 02.3 Hz), and no case exceeding 2.3 Hz deviation (Table I). These minor variations fall well within the typical 3 Hz bandwidth employed for subject-specific beta filtering and are unlikely to compromise burst detection sensitivity or specificity. This high level of agreement validates the sliced computation algorithm as a reliable and efficient real-time peak frequency estimation method in resource-constrained implantable systems.

**TABLE I:** Peak Frequency Estimation Result

Subject	Proposed Algorithm (Hz)	Long-window FFT (Hz)
1	32	32.5394
2	34	31.7535
3	30	29.9988
4	28	28.6484
5	29	29.6021
6	28	28.4576
7	32	32.3257
8	32	31.5475
9	28	27.8091

Mean absolute difference 0.61 0.64 Hz.

### B. Computational Efficiency of the Proposed Peak Estimation Method

Computational complexity analysis focused primarily on FFT operations, the most computationally intensive step in the processing pipeline. The evaluation used representative 256-second LFP datasets sampled at 1 kHz, with power-of-two parameters selected to facilitate hardware implementation. The conventional approach exhibits *O*(*N* log_2_ *N*) complexity (*N* 262144 samples, log_2_ *N* 18). Our sliced method achieves *O*(*N* log_2_ *L*) complexity (*L* 1024 points per segment, log_2_ *L* 10), corresponding to a 44.4 reduction in computational operations.

For implantable applications targeting neural signals below 35 Hz, a downsampled configuration (128 Hz sampling, 128-point FFT) was evaluated. This configuration achieves a 53.3 reduction in complexity (log_2_ *L* 7 vs. log_2_(32768) 15) while maintaining 1 Hz spectral resolution.

Table II summarizes the computational complexity associated with peak frequency estimation under different processing configurations. The Standard configuration corresponds to the original 1 kHz sampling rate with 1024-point FFT windows, whereas the Downsampled configuration employs 128 Hz sampling with 128-point FFTs, reflecting a hardware-oriented design optimized for beta-band signals below 35 Hz. The Statistical Window column indicates the equivalent total analysis duration (*N*) used by the conventional long-window FFT approach for comparison. The reported relative complexity reduction is normalized with respect to the logarithmic FFT term, highlighting the impact of reducing the effective FFT length from *N* to *L*.

**TABLE II:** Computational Complexity Comparison for Peak Frequency Estimation

Configuration	Sliced Algorithm Complexity (log_2_ L)	Statistical Window (Second)	Conventional Complexity (log_2_ N)	Relative Complexity Reduction ()
Standard (FFT Window N 1024)	10	64	16	37.5
		128	17	41.2
		256	18	44.4
		512	19	47.4
Downsampled (FFT Window N 128)	7	64	13	46.2
		128	14	50.0
		256	15	53.3
		512	16	56.3

Memory requirements showed even greater improvement: our method requires only 1-second data buffers compared to the conventional 256-second buffers, representing a 256-fold reduction that scales proportionally with recording duration. Detailed complexity comparisons are summarized in Table II. These significant computational and memory optimizations make our algorithm well-suited for low-power, resource-constrained real-time processing in implantable devices

### C. Burst Amplitude Consistency Between Methods

We evaluated the consistency of burst amplitude measurements derived from our proposed frequency-domain power metric and those obtained from the conventional time-domain amplitude envelope method. Both approaches were applied to standardized one-second, non-overlapping segments of the ECoG recordings to ensure a fair comparison.

**Fig. 2.**
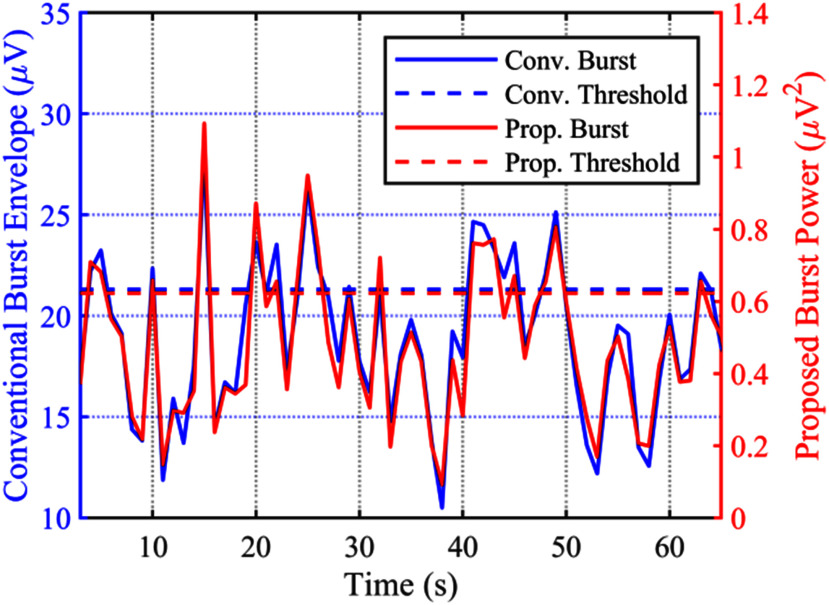
Temporal alignment of the conventional burst envelope and the proposed burst power. The graph displays the temporal evolution of the conventional burst envelope and the proposed band power, alongside their respective burst detection thresholds. This illustrates the dynamic performance of both methods. The example is taken from rat6 in the ECoG dataset.

**Fig. 3.**
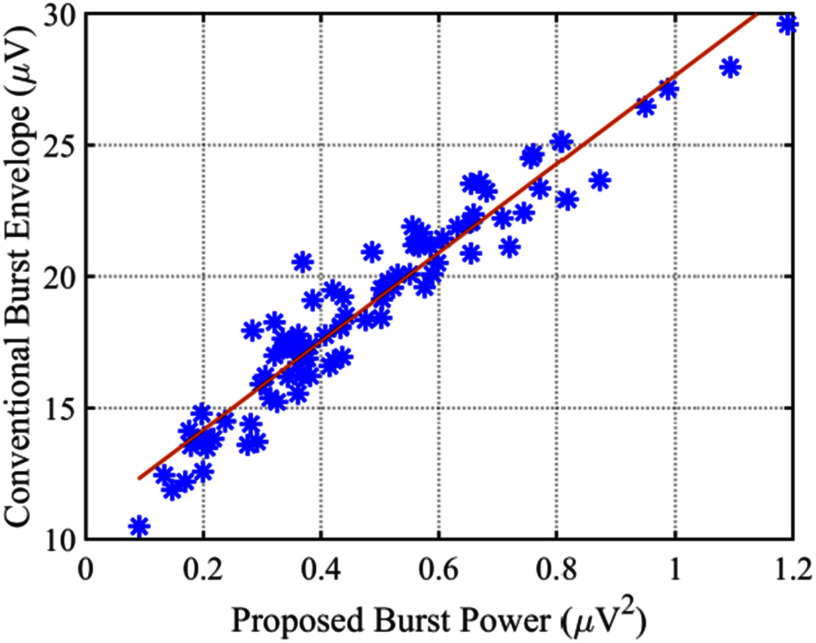
Correlation between conventional burst envelope and proposed burst power. A scatter plot illustrating the relationship between the conventional burst envelope (Y-axis) and the proposed band power (X-axis), with the red line indicating the linear regression fit. Data were obtained from rat6 (ECoG), as described in the methods section. The spearman correlation coefficient in this case was 0.95 (p < 0.001).

Spearman correlation analysis across all nine datasets revealed a strong positive correlation (mean 0.89 0.06; all p < 0.001), indicating that the frequency-domain method reliably captures burst intensity consistent with conventional amplitude-based analysis. The individual Spearman coefficients are summarized in Table III, demonstrating consistent agreement across all subjects. Other performance metrics in the table are discussed in Section D. Fig. 2 illustrates the temporal alignment of burst amplitude profiles from both methods in a representative LFP segment. At the same time, Fig. 3 presents the corresponding scatter plot confirming the high correlation.

**TABLE III:** Burst Feature Comparison Between Proposed and Conventional Method

Subject	Spearman rho	Cohens kappa	F1-score	Recall
1	0.85477	0.6958	0.88	0.8462
2	0.78536	0.7324	0.8571	0.8571
3	0.93323	0.7656	0.7857	0.7857
4	0.81995	0.564	0.6897	0.7692
5	0.90516	0.8467	0.9032	0.9333
6	0.95158	0.8467	0.8889	0.9231
7	0.92806	0.7058	0.9091	0.8333
8	0.94651	0.6729	0.8667	0.8667
9	0.92743	0.7918	0.8667	0.9286
Mean SD	0.89 0.06	0.74 0.09	0.85 0.07	0.86 0.06

Quantitative metrics: Spearman rho for burst amplitude; Cohens kappa for burst classification; F1-score and Recall for burst event.

This strong agreement validates the frequency-domain power metric as a robust surrogate for real-time burst detection in resource-constrained implantable systems.

### D. Comparison of Gaussian-based Threshold Estimation Results

We evaluated the performance of the online Gaussian-based threshold approximation and its impact on burst classification. Gaussian-derived thresholds were converted to equivalent percentiles for threshold accuracy assessment and compared against target values. Across all datasets (n 9), the mean absolute deviations were 2.97 3.22 percentage points for the 75th percentile (coefficient 0.675), 5.03 2.97 for the 65th percentile (coefficient 0.385), and 2.80 2.17 for the 85th percentile (coefficient 1.036). These slight deviations, achieved without requiring historical data buffers, fall within an acceptable tolerance range for system-level burst detection, defined here as the regime in which classification accuracy and agreement metrics remain robust for adaptive control logic. As detailed in the following analysis, these deviations do not compromise the final burst detection performance.

System-level burst detection performance was assessed through classification agreement and event-level accuracy. Binary burst/non-burst classification agreement yielded a Cohens kappa of 0.74 0.09, indicating substantial agreement with the reference method [27]. Event-level detection achieved an F1-score of 0.85 0.07 and recall of 0.86 0.06, with true positives defined by temporal co-occurrence within a one-window tolerance. Detailed metrics for all datasets are summarized in Table III. The observed high agreement and detection accuracy further confirm that the Gaussian-based threshold deviations remain within a practically acceptable range, preserving reliable burst event identification for closed-loop DBS control.

These results, combined with peak frequency estimation accuracy, computational efficiency, and amplitude consistency validation, confirm that the proposed system achieves strong concordance with conventional burst detection methods while delivering significant reductions in memory and computational requirements. The algorithm is therefore well-suited for real-time closed-loop DBS deployment in implantable systems.

### E. Estimated Computational and Memory Resource Usage

To provide quantitative evidence of hardware suitability without requiring silicon-level implementation, we performed a first-order, algorithm-level resource estimation under representative implantable system constraints. We evaluated computational and memory requirements under a representative fixed-point configuration (16-bit input resolution and 32-bit internal processing). Assuming a downsampled rate of 128 Hz and non-overlapping 1-s analysis windows over a 256-s statistical period, the conventional approach requires buffering the full signal history for spectral analysis and percentile-based thresholding, resulting in an estimated memory footprint of approximately 192 kB. In contrast, the proposed sliced approach processes data on a per-window basis, reducing the active memory requirement to less than 1 kB, including input and spectral processing buffers. In addition, the proposed method replaces the sorting operation required for percentile threshold estimation with a streaming Gaussian-based update, reducing the threshold adaptation cost from *O*(*N* log_2_ *N*) to constant-time arithmetic operations. Under these conditions, the proposed approach achieves an estimated 53.3 reduction in FFT-related computational load and more than two orders of magnitude reduction in memory usage, highlighting its suitability for resource-constrained embedded implementations.

It is important to clarify the difference in output resolution between the conventional time-domain method, which produces a burst envelope at the sampling rate (128 Hz), and the proposed frequency-domain approach, which operates on a window-based representation (1 Hz). In conventional adaptive DBS systems, although burst-related features are computed at the sampling rate, stimulation control schemes typically rely on temporally smoothed signals, such as moving-average filtering over hundreds of milliseconds, to ensure stable control updates.

Motivated by this commonly adopted temporal smoothing practice, the proposed sliced approach represents burst activity by integrating spectral power over a 1-s window. This formulation aligns the feature representation with the effective temporal resolution used for control, while providing a more compact computational abstraction. Importantly, this design choice does not question the validity or effectiveness of established time-domain methods, which have been extensively validated in clinical studies, but instead reflects a different representation level selected for computational efficiency.

Regarding threshold adaptation, the proposed Gaussian-based update involves a square-root operation for variance estimation. Although this operation is computationally nontrivial, it is performed only once per statistical window. From an implementation perspective, such infrequent updates allow this computation to be amortized over time, resulting in a limited contribution to the overall computational load when compared with continuously updated envelope extraction and filtering operations in conventional pipelines.

A summary of the estimated computational and memory requirements for both approaches is provided in Table IV. The reported values are derived from algorithmic operation counts and buffer sizes under the stated assumptions, and are intended to illustrate relative resource trends rather than measured hardware performance.

**TABLE IV:** Estimated Algorithm-Level Computational and Memory Resource Usage for Beta Burst Detection

**Resource Metric**	**Conventional Method** [8]	**Proposed Method**	**Algorithmic Insight**
**SRAM Memory Footprint**	192 KB	< 1 KB (0.8 KB)	240 reduction
Raw Data Buffer	64 KB (full history)	0.25 KB (1-s slice)	
Processing Buffer	128 KB (FFTenvelope)	0.5 KB (FFTpower)	
Burst Feature Extraction	Sample-wise envelope	Window-based integration	Representation-level abstraction
Output Update Rate	128 Hz (sample-wise)	1 Hz (window-wise)	Temporal aggregation
Burst Filtering	164 kMACs (2nd-order IIR)	Integrated in FFT	Domain-level aggregation
Temporal Smoothing	Moving average low-pass filter	Implicit in windowing	Integrated operation
Spectral Analysis	491 kCMACs (*N* log_2_ *N*)	229 kCMACs (*N* log_2_ *L*)	Short-window FFT
Threshold Adaptation	*O*(*N* log_2_ *N*) (Sorting)	*O*(*1*) (Gaussian update)	Streaming statistics
Square-root Operation ()	NA	Once per statistical window	Low update frequency

Table IV. Estimated algorithm-level computational and memory resource usage under a 256-s statistical window with a sampling rate of 128 Hz, assuming 16-bit input resolution and 32-bit internal processing. For the proposed method, non-overlapping 1-s analysis windows (*L* 128 samples) are used. Operation counts are estimated based on algorithmic complexity and update frequency and are reported for comparative purposes rather than measured hardware performance. Computational intensity is quantified using MAC (multiplyaccumulate) operations for real-valued filtering and CMAC (complex multiplyaccumulate) operations for FFT-based spectral analysis. Micro-architectural details (e.g., FFT formulations, filter realizations, coefficient quantization, and bit-width-dependent optimizations) are not explicitly modeled, as they may affect absolute values but do not alter the relative computational trends reported in this table.

## IV. Discussion

This study addresses a fundamental limitation hindering the practical implementation of closed-loop deep brain stimulation (DBS) in implantable systems: the high computational complexity and memory requirements of existing beta burst detection methods. While effective for offline analysis, conventional approaches are incompatible with long-term, battery-operated devices. Our algorithmic innovations demonstrate that sophisticated neural signal processing can be achieved through substantial computational optimization without compromising detection accuracy.

### A. Algorithmic Contributions and Computational Advances

This study presents an algorithmic framework designed to enable real-time adaptive processing in implantable DBS systems through substantial computational optimization. The proposed sliced computation algorithm for peak frequency estimation, leveraging a mode-based approach, significantly reduces logarithmic complexity by 44.4 in standard configurations (log_2_ *N* 18 to log_2_ *L* 10) and 53.3 in downsampled configurations (log_2_ *N* 15 to log_2_ *L* 7) compared to conventional methods. These computational savings directly translate to reduced power consumption, critical for implantable devices where energy efficiency dictates operational lifespan.

A key paradigm shift is our frequency-domain approach with shared FFT computations. Unlike conventional methods that perform independent operations, our dual-unit architecture leverages common computational resources for continuous burst detection and parallel model updating. This approach improves efficiency and ensures consistency across processing stages, as evidenced by the strong correlation ( 0.89 0.06) between frequency-domain power metrics and conventional time-domain amplitude measurements.

### B. Real-time Parameter Adaptation and Clinical Reliability

A critical innovation is the implementation of online adaptive methods for peak frequency tracking and threshold estimation. The mode-selection algorithm continuously updates peak frequency estimates, while the Gaussian-based approximation dynamically adjusts detection thresholds based on evolving signal characteristics. This eliminates the need for large historical buffers or offline recalibration, enabling autonomous long-term operation. Validation confirmed high peak frequency concordance (absolute difference 1 Hz in 88.9 of cases) and reasonable threshold accuracy (mean absolute deviation of 2.97 3.22 percentage points for the 75th percentile). The strong agreement metrics (Cohens kappa 0.74 0.09, F1-score 0.85 0.07) validate the computational robustness and functional fidelity of our improved approach. These performance levels exceed minimum thresholds required for biomedical signal processing applications, confirming that computational optimizations preserve essential physiological information. At the same time, the mode-selection approach enhances peak frequency robustness to transient artifacts.

### C. Hardware Implementation Considerations

The proposed system was designed with hardware feasibility as a primary consideration. The design minimizes memory access by employing one-second, non-overlapping FFT windows and enables pipeline-friendly processing. The sliced computation architecture and online statistical threshold estimation significantly reduce memory footprint and computational burden. These improvements facilitate real-time deployment on low-power embedded platforms, including application-specific integrated circuits (ASICs), digital signal processors (DSPs), and commercially available neuromodulation platforms such as the Medtronic Summit RC+S system [21].

Unlike conventional real-time burst detection strategies that typically rely on continuous time-domain filtering followed by envelope extraction and memory-intensive percentile sorting using long historical buffers, the proposed framework operates in the frequency domain with a sliced architecture. This approach avoids the need for large data buffers and sorting operations, thereby minimizing the dominant memory constraints encountered in implantable hardware, as detailed in the quantitative resource analysis in Section III-E.

### D. Limitations

Several limitations should be acknowledged. First, our evaluation focused on acute recordings from a 6-OHDA rat model using cortical ECoG signals, which may not fully represent the long-term neural dynamics observed in chronic human DBS implants. Compared with rat ECoG recordings obtained under controlled experimental conditions, human STN-LFP signals may exhibit greater variability and nonstationarity associated with medication status, behavioral context, and circadian modulation. Previous clinical studies have reported that human STN-LFP activity may exhibit dynamic shifts between low-beta and high-beta frequency components across different physiological states. To facilitate future clinical translation, the proposed framework adopted a 128-Hz sampling rate and a 1-Hz frequency resolution selected with consideration of the spectral characteristics of human STN-LFP signals. Because adaptive DBS implementations commonly employ subject-specific filtering windows spanning several hertz around the identified peak frequency (e.g., 3 Hz), a 1-Hz tracking granularity is expected to remain sufficient for maintaining dynamically shifting pathological beta activity within the targeted therapeutic band. Nevertheless, further validation using chronic human STN-LFP datasets will be necessary to characterize algorithmic performance under realistic clinical spectral variability.

Second, while Gaussian-based threshold approximation performed well on average, accuracy varied across datasets. The underlying assumption of normally distributed beta power may not universally hold across all patient populations. However, similar to percentile-based thresholds in reference algorithms, our method provides flexible parameterization supporting reasonable threshold adjustment without strict distributional assumptions.

Third, the one-second window length compromises spectral resolution and computational efficiency. Non-overlapping windows result in once-per-second burst detection, potentially limiting sensitivity to brief bursts. However, our approach remains consistent with existing biomarker literature reporting detection intervals ranging from 20ms to 1 minute, and sensitivity could be enhanced through threshold parameter tuning [10], [28], [29]. Furthermore, the window-based integration inherently provides temporal smoothing beneficial for preventing rapid control fluctuations (chatter) in closed-loop operation.

Finally, it is important to clarify that this work focuses on algorithmic design and offline validation. While the presented complexity analysis and memory profiling provide a theoretical basis for hardware deployment, actual implementation would require circuit-level design, power characterization, and validation on implantable or research-oriented bidirectional platforms (e.g., Medtronic Summit RC+S or similar systems), which are beyond the scope of this study. Such evaluations would provide additional evidence regarding processing latency, energy consumption, and clinical equivalence under realistic deployment conditions. Nevertheless, the demonstrated computational efficiency and causal streaming architecture establish a practical foundation for future hardware-oriented investigations and translational studies.

### E. Clinical Impact and Future Directions

This algorithmic framework provides a robust algorithm-level computational foundation for next-generation adaptive neuromodulation systems. The ability to perform real-time parameter updating, combined with reduced computational requirements, enables more sophisticated closed-loop control strategies that were previously impractical due to hardware limitations.

Recent fully integrated closed-loop neuromodulation systems-on-chip, such as NeuralTree [18], have demonstrated versatile on-chip signal processing and classification capabilities under stringent implantable constraints. In contrast, the present work focuses on optimizing the beta-burst feature extraction engine itself, which constitutes a key computational bottleneck in adaptive DBS pipelines rather than a complete closed-loop SoC. From this perspective, the proposed frequency-domain architecture complements higher-level system designs by providing a resource-efficient spectral processing core that can be incorporated into diverse adaptive neuromodulation platforms.

Although alternative adaptive DBS paradigms, including machine-learning-based tremor detection [30] and phase-specific stimulation [9], have shown promise in specific clinical contexts, beta burst activity remains a well-established biomarker for rigidity and bradykinesia. Accordingly, the proposed amplitude-based framework is complementary to phase-locked or multimodal strategies, underscoring the importance of aligning biomarker selection and control schemes with symptom-specific and system-level requirements.

Integration with advanced signal processing techniques like spectral coherence analysis or multisite signal fusion becomes feasible through shared FFT computations. From a computational perspective, this work represents a significant step toward fully adaptive, long-term neuromodulation therapies that can respond dynamically to evolving neural dynamics and therapeutic needs within the stringent constraints of implantable devices. Furthermore, future work will include detailed power modeling and hardware prototyping on ultra-low-power embedded platforms to validate clinical feasibility. Machine learning approaches could refine patient-specific parameter adaptation, and multi-channel processing could enhance detection accuracy.

## V. Conclusion

This study demonstrates that substantial computational optimization of beta burst detection can be achieved while maintaining high concordance with standard clinical benchmarks. The combination of sliced computation, frequency-domain processing, and adaptive parameter estimation provides a robust framework for real-time neural biomarker extraction in resource-constrained implantable systems. By maintaining strong correlation with established methods ( > 0.85) while achieving significant computational improvements, our approach makes closed-loop DBS implementation computationally more feasible, representing a valuable contribution toward fully adaptive, long-term neuromodulation therapies.

## Supplementary Materials

Supplementary Materials
