# Supplementary material for: An Improved Beta Burst Extraction for Chip-Based Deep Brain Stimulation With Real-Time Model Updating
Source: IEEE Open J Eng Med Biol. 2026 May 25;7:214–22. doi: 10.1109/OJEMB.2026.3695567 (PMC13278738; doi:10.1109/OJEMB.2026.3695567)
Supplement: Supplementary Materials [file supp1-3695567.pdf]

An Improved Beta Burst Extraction for Chip-based Deep Brain Stimulation with Real-Time Model Updating

Supplementary Materials

Yi-Huan Ou-Yang, Hsiao-Chun Lin, Chi-Wei Huang, Chung-Yu Wu, *Life Fellow, IEEE*, Ming-Dou Ker, *Fellow, IEEE*, and Chen-Yi Lee

I. Detailed Experimental Protocols

A. *Animal Subjects and Husbandry*

Male Sprague-Dawley rats, weighing between 220 and 260 g, were procured from BioLasco Taiwan Co., Ltd. The animals were maintained at the Laboratory Animal Center on the Boai Campus of National Yang Ming Chiao Tung University. To ensure social welfare, rats were co-housed in pairs within standard cages under a strictly controlled environment: ambient temperature of 22 ± 2℃, relative humidity maintained between 50% and 70%, and a 12-hour light/dark cycle (lights on at 07:00). All subjects had unrestricted access to standard rodent chow and water. The experimental protocols strictly adhered to the guidelines approved by the Institutional Animal Care and Use Committee (IACUC) under protocol number NCTU-IACUC-110033.

B. *Surgical Induction of Parkinsonian Phenotype*

Prior to surgical intervention, animals underwent a mandatory acclimatization period of at least one week. A total of 14 rats were utilized for this phase. Anesthesia was induced via intraperitoneal (i.p.) injection of a mixture containing Tiletamine-zolazepam (50 mg/kg) and xylazine (10 mg/kg). Following sterilization with alcohol and povidone-iodine, a longitudinal incision was made to expose the calvarium.

The 6-hydroxydopamine (6-OHDA) solution was prepared by dissolving the neurotoxin in sterile saline containing 0.1% ascorbic acid to a final concentration of 3 μg/μL. A burr hole was drilled above the right medial forebrain bundle (MFB). Using a Hamilton microsyringe mounted on a stereotaxic microdrive, 3 μL of the solution was infused at the following coordinates relative to bregma: AP -4.2 mm, ML +1.8 mm, and DV -7.6 mm. To prevent backflow, the needle remained in situ for 5 minutes post-injection before being slowly withdrawn. The incision was closed using 4-0 nylon sutures, and Carprofen (5 mg/kg, i.p.) was administered for postoperative analgesia.

Three weeks post-lesioning, dopaminergic depletion was assessed via the apomorphine rotation test (0.5 mg/kg, i.p.). Subjects exhibiting vigorous contralateral rotations (>6 turns/min) within 20 minutes were classified as successfully induced models. Animals failing to meet this criterion were euthanized using CO_2 asphyxiation.

C. *Cortical Electrode Implantation and Signal Acquisition*

The nine validated Parkinsonian rats underwent a second stereotaxic procedure under the identical anesthetic protocol described above. The surgical arrangement of the sensors followed a specific spatial configuration to capture motor-related potentials (Fig. S1a). Specifically, a stainless-steel screw (Ø 1.4 mm) was threaded into the skull over the right motor cortex (AP +3.0 mm, ML +3.0 mm relative to bregma), with the tip contacting the dura mater without penetrating the parenchyma. A reference electrode was secured over the cerebellar region to ensure stable differential recording. The entire electrode assembly was rigidly stabilized using biocompatible dental acrylic cement to maintain long-term signal integrity.

Following a one-week recovery period, neural data were collected from awake, freely moving animals in a dedicated observation enclosure (Fig. S1b). This setup facilitated the collection of high-fidelity ECoG data while the subjects were in an active state, ensuring that the recorded neural dynamics represented the typical pathological state of the Parkinsonian model without the interference of sedative agents. ECoG signals underwent 1000-fold amplification and analog filtering (0.1–500 Hz bandpass) via an A-M Systems Model 1800 amplifier. Data digitization was performed at a sampling rate of 1 kHz using a PowerLab 8/30 system (ADInstruments). For each subject, a 3-minute continuous recording was obtained. During offline processing, visual inspection was used to exclude epochs containing gross movement artifacts, resulting in a consistent 90-second stable baseline for subsequent analytical procedures.

II. Signal Processing and Beta Burst Detection

To extract the pathological biomarkers relevant to Parkinsonian motor deficits, the raw ECoG data underwent spectral decomposition. Fig. S2 illustrates the signal processing pipeline applied to a representative recording session using the proposed sliced computation framework. The raw neural oscillations (Fig. S2, top panel) were filtered to isolate the beta

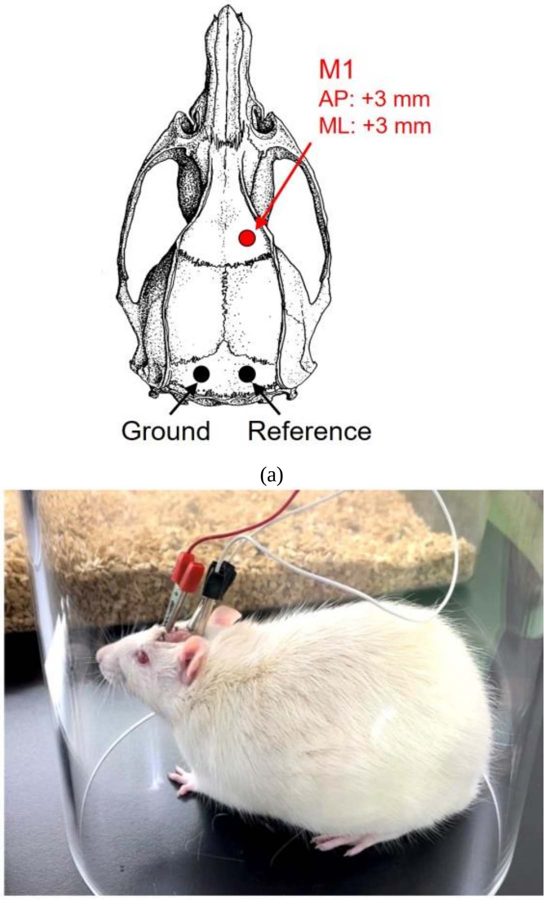

(a)

(b)

Fig. S1. Electrode configuration and experimental recording environment. (a) Schematic representation of the rat cranium illustrating the precise coordinates for electrode placement. The recording electrode is targeting the primary motor cortex (M1) at AP: +3.0 mm and ML: +3.0 mm relative to bregma. Reference and ground electrodes are positioned posteriorly over the cerebellum to ensure stable differential recording. (b) Representative photograph of a conscious, unrestrained Sprague-Dawley rat during an electrocorticography (ECoG) session. The animal is situated within a transparent recording chamber, allowing for the acquisition of neural oscillations during spontaneous behavior.

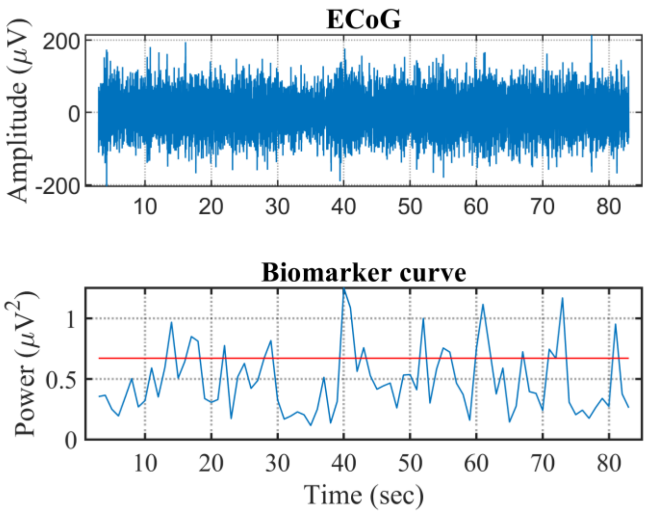

Fig. S2. Representative ECoG time-series and derived beta-band biomarker dynamics. (Top) A segment of raw electrocorticography (ECoG) signals recorded from the motor cortex of a freely moving Parkinsonian rat. The trace exhibits characteristic oscillatory activity associated with the 6-OHDA-induced lesion. (Bottom) The corresponding time-varying beta burst power curve (P_{beta}) generated using the proposed frequency-domain extraction method. The horizontal red line demarcates the burst detection threshold, defined at the 75th percentile of the power distribution. Suprathreshold events (where the blue curve exceeds the red line) are identified as beta bursts, serving as the temporal trigger for the closed-loop control algorithm.

frequency band. Subsequently, the proposed frequency-domain biomarker extraction was applied to generate the time-varying beta burst power curve (P_{beta}) (Fig. S2, bottom panel). To identify pathological burst events, a static threshold was established at the 75th percentile of the recording's power distribution. This thresholding approach allows for the real-time identification of high-power beta events, which are hypothesized to correlate with bradykinesia and rigidity in the rodent model.
